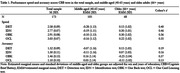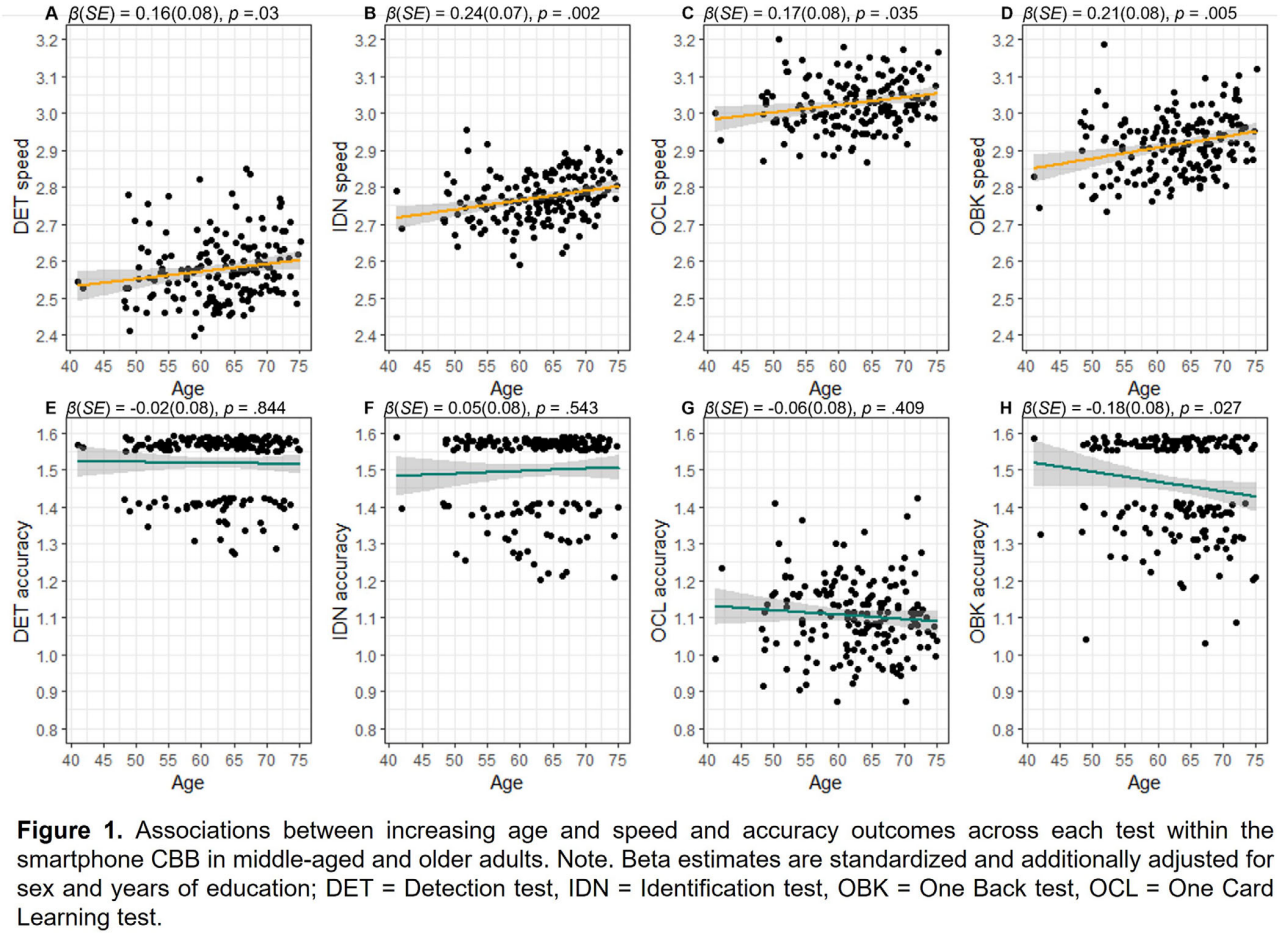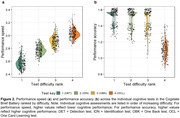# Acceptability and validity of the smartphone‐administered version of the Cogstate Brief Battery in middle‐aged and older adults of the Healthy Brain Project

**DOI:** 10.1002/alz.091946

**Published:** 2025-01-03

**Authors:** Hannah Cummins, Lisa Bransby, Amy Fredrickson, Gabriel Stellman, Paul Maruff, Chris J. Edgar, Yen Ying Lim

**Affiliations:** ^1^ Turner Institute for Brain and Mental Health, Monash University, Melbourne, VIC Australia; ^2^ Cogstate Ltd, Melbourne, VIC Australia; ^3^ Cogstate Ltd, Chicago, IL USA; ^4^ Cogstate Ltd., Melbourne, VIC Australia; ^5^ The Florey Institute of Neuroscience and Mental Health, The University of Melbourne, Parkville, VIC Australia; ^6^ Cogstate Ltd., London United Kingdom; ^7^ Turner Institute for Brain and Mental Health, School of Psychological Sciences, Monash University, Melbourne, VIC Australia

## Abstract

**Background:**

Remote and unsupervised administration of neuropsychological tests may increase opportunities to understand cognition in everyday life compared to in‐clinic assessments. The technological sophistication and widespread use of smartphones now allows this platform to be used for remote neuropsychological testing. However, it is important to ensure that the validity of neuropsychological tests extends to remote and unsupervised administration. As the Cogstate Brief Battery (CBB) has been validated for remote use on computers, the aim of this study was to now examine whether the acceptability and validity holds when administered via smartphone to cognitively unimpaired (CU) middle‐aged and older adults.

**Method:**

CU adults (n = 173) aged 41‐75 years (M(SD) = 62.72 (7.18)) enrolled in the Healthy Brain Project completed the smartphone CBB remotely. The mean speed and accuracy of performance on the Detection (DET, psychomotor function), Identification (IDN, attention), One Back (OBK, working memory) and One Card Learning (OCL, visual learning) tests were used to define performance. Acceptability was determined by the percentage of participants completing the full CBB. Relationships between CBB performance with age and test difficulty were described to inform validity. Relationships between objective CBB performance and subjective ratings of how participants felt about their overall performance, obtained via survey, were also explored.

**Result:**

100% of participants completed the smartphone CBB. Increasing age was associated with slower performance on all tests (Figure 1a‐1d), and reduced accuracy on the OBK (Figure 1h). Older adults (66+ years) performed slower than middle‐aged adults (40‐65 years) on all CBB tests, and with less accuracy on OBK (Table 1). Performance speed across all tests slowed with increasing test difficulty (Figure 2). Whilst a ceiling effect was observed for accuracy of simpler tests (DET, IDN, OBK), accuracy was lowest for the most difficult test (OCL) (Figure 2). Subjective ratings of overall CBB performance was significantly, albeit weakly, correlated with OCL accuracy (*r* = 0.23, *p* = .003).

**Conclusion:**

The results support the acceptability and validity of the remote, unsupervised administration of the smartphone CBB in CU middle‐aged and older adults. These results provide a foundation for gaining further understanding of the performance of the smartphone CBB in diverse and impaired populations.